# Reference Ranges for Bone Mineral Density and Prevalence of Osteoporosis in Vietnamese Men and Women

**DOI:** 10.1186/1471-2474-12-182

**Published:** 2011-08-10

**Authors:** Lan T Ho-Pham, Uyen D T Nguyen, Hoa N Pham, Nguyen D Nguyen, Tuan V Nguyen

**Affiliations:** 1 Department of Internal Medicine, Pham Ngoc Thach University of Medicine, 86/2 Thanh Thai St, Ward 12, District 10, Ho Chi Minh City, Vietnam; 2 Department of Rheumatology, People's Hospital 115, 88 Thanh Thai Street, Ward 12, District 10, Ho Chi Minh City, Vietnam; 3 Department of Nuclear Medicine, Cho Ray Hospital, 201B Nguyen Chi Thanh Street, District 5, Ho Chi Minh City, Vietnam; 4 Osteoporosis and Bone Biology Program, Garvan Institute of Medical Research, 384 Victoria Street, Sydney NSW 2010, Australia; 5 St Vincent's Clinical School, Victoria Street, Sydney NSW 2010, Australia; 6School of Public Health and Community Medicine, University of New South Wales, NSW 2052, Australia

**Keywords:** *reference range*, *bone mineral density*, *osteoporosis*, *women*, *men*

## Abstract

**Background:**

The aim of this study was to examine the effect of different reference ranges in bone mineral density on the diagnosis of osteoporosis.

**Methods:**

This cross-sectional study involved 357 men and 870 women aged between 18 and 89 years, who were randomly sampled from various districts within Ho Chi Minh City, Vietnam. BMD at the femoral neck, lumbar spine and whole body was measured by DXA (Hologic QDR4500). Polynomial regression models and bootstraps method were used to determine peak BMD and standard deviation (*SD*). Based on the two parameters, we computed T-scores (denoted by *T*_VN_) for each individual in the study. A similar diagnosis was also done based on T-scores provided by the densitometer (*T*_DXA_), which is based on the US White population (NHANES III). We then compared the concordance between *T*_VN _and *T*_DXA _in the classification of osteoporosis. Osteoporosis was defined according to the World Health Organization criteria.

**Results:**

In post-menopausal women, the prevalence of osteoporosis based on femoral neck *T*_VN _was 29%, but when the diagnosis was based on *T*_DXA_, the prevalence was 44%. In men aged 50+ years, the *T*_VN_-based prevalence of osteoporosis was 10%, which was lower than *T*_DXA_-based prevalence (30%). Among 177 women who were diagnosed with osteoporosis by *T*_DXA_, 35% were actually osteopenia by *T*_VN_. The kappa-statistic was 0.54 for women and 0.41 for men.

**Conclusion:**

These data suggest that the *T-*scores provided by the Hologic QDR4500 over-diagnosed osteoporosis in Vietnamese men and women. This over-diagnosis could lead to over-treatment and influence the decision of recruitment of participants in clinical trials.

## Background

Osteoporosis and its consequence of fragility fracture represent a major public health problem not only in developed countries, but in developing countries as well [1]. The number of fractures in Asia is higher than that in European countries combined. Of all the fractures in the world, approximately 17% was found to occur in Southeast Asia, 29% in West Pacific, as compared to 35% occurring in Europe [2]. However, the prevalence of and risk factors for osteoporosis in Asian populations have not been well documented. Part of the problem is due to the lack of well-defined criteria for the diagnosis of osteoporosis in Asian men and women.

Currently, the operational definition of osteoporosis is based on a measurement of bone mineral density (BMD), which is the most robust predictor of fracture risk [3,4]. BMD of an individual is often expressed in terms of its peak level and standard deviation to yield a *T*-score. The two parameters (i.e., peak BMD level and standard deviation) are commonly derived from a well characterized population of young individuals [5]. An individual's *T*-score is actually the number of standard deviations from the peak BMD achieved during the age of 20 and 30 years [6,7]. However, previous studies have suggested that peak BMD is different among ethnicities and between men and women [8,9]. Therefore, the diagnosis of osteoporosis should ideally be based on sex- and ethnicity-specific reference range [10,11].

Dual-energy × ray absorptiometry (DXA) is considered the gold standard method for measuring BMD [6]. In recent years, DXA has been introduced to many Asian countries, including Vietnam, and is commonly used for the diagnosis of osteoporosis and treatment decision. In the absence of sex-specific reference data for local population, most doctors used the T-scores provided by the densitometer as a referent value to make diagnosis for an individual. However, it is not clear whether the reference data base used in the derivation of T-scores in these densitometers is appropriate for a local population. We hypothesize that there is considerable discrepancy in the diagnosis of osteoporosis between reference data. The present study was designed to test the hypothesis, by determining reference range of peak bone density for an Asian population, and then comparing the concordance between a population-specific T-score and the DXA-based T-score in the diagnosis of osteoporosis.

## Methods

### Study design and participants

The study was designed as a cross-sectional investigation, with the setting being Ho Chi Minh City, a major city in Vietnam. The research protocol and procedures were approved by the Scientific Committee of the People's Hospital 115 and Pham Ngoc Thach University of Medicine. All volunteer participants were provided with full information about the study's purpose and gave informed consent to participate in the study, according to the principles of medical ethics of the World Health Organization.

We used simple random sampling technique for identifying potential participants. We approached community organizations, including church and temples, and obtained the list of members, and then randomly selected individuals aged 18 or above. We sent a letter of invitation to the selected individuals. The participants received a free health check-up, and lipid analyses, but did not receive any financial incentive. No invited participants refused to participate in the study.

Participants were excluded from the study if they had diseases deemed to affect to bone metabolism such as hyperthyroidism, hyperparathyroidism, renal failure, malabsorption syndrome, alcoholism, chronic colitis, multi- myeloma, leukemia, and chronic arthritis.

### Measurements and data collection

Data collection was done by trained research doctors and nurses using a validated questionnaire. The questionnaire solicited information, including anthropometry, lifestyle factors, dietary intakes, physical activity, and clinical history. Anthropometric parameters including age, weight, standing height were obtained. Body weight was measured on an electronic scale with indoor clothing without shoes. Height was determined without shoes on a portable stadiometer with mandible plane parallel to the floor. Each participant was asked to provide information on current and past smoking habits. Smoking was quantified in terms of the number of pack-years consumed in each ten-year interval age group. Alcohol intake in average numbers of standard drinks per day, at present as well as within the last 5 years, was obtained. Clinical data including blood pressure, pulse, and reproductive history (i.e. parity, age of menarche, and age of menopause), medical history (i.e. previous fracture, previous and current use of pharmacological therapies) were also obtained.

### Bone mineral density

Areal BMD was measured at the lumbar spine (L2-L4), femoral neck, and whole body using a Hologic QDR 4500 (Hologic Corp, Madison, WI, USA). The short-term ***in vivo *precision **expressed as the coefficient of variation was 1.8% for the lumbar spine and 1.5% for the hip. The machine was standardized by standard phantom before each measurement.

The densitometer provided a T-score for each measured site. In this paper, the T-score is referred to as *T_DXA_*. We used the WHO criteria to categorize *T_DXA _*into three groups: *osteoporosis *if the T-score is equal to or lower than -2.5; *osteopenia *if T-score is between -1 and -2.5; and *normal *if T-score is equal or greater than -1.

### Determination of reference range

In this analysis, we made use of the functional relationship between BMD and age to construct a reference range. A series of polynomial regression models (up to the third degree) were fitted to femoral neck, total hip and lumbar spine BMD as a function of age as follows: BMD = α + β_1_(age) + β_2_(age)^2 ^+ β_3_(age)^3^, where α is the intercept, β_1_, β_1_, and β_3 _are regression parameters, which were estimated from the observed data. Reduced models (i.e., quadratic and linear models) were considered, and the "final" model was chosen based on the Akaike Information Criterion (AIC). Peak BMD (*p*BMD) and ages at which it was reached were then estimated from the final model. Ninety-five percent confidence intervals (95% CI) for *p*BMD and ages of *p*BMD were determined by the bootstrap (resampling) method. The analysis was performed with R statistical software [12].

Based on the parameters in the polynomial regression models, we determined the means of peak BMD and standard deviation (SD) for spine and femoral neck BMD. Using the two parameters, we calculated the T-score for each individual (denoted by *T*_VN_), and used the WHO criteria to classify the T-score into three groups, namely, osteoporosis, osteopenia, and normal. The concordance between *T*_DXA _and *T*_VN _was then assessed by the kappa statistic.

## Results

In total, 1227 individuals (357 men and 870 women) aged 18 years or older participated in the study. In this sample, 58.5% of men and 51% of women were at age 50+ years, respectively. As expected, BMD in men was higher than in women by ~12% at femoral neck and by ~7% at lumbar spine (Table 1).

**Table 1:** Characteristics of participants

Variable	Men	Women	P-value
	(n = 357)	(n = 870)	
Age (years)	43.5 (18.8)	48.6 (16.5)	< 0.0001
Weight (kg)	62.1 (9.6)	52.4 (8.6)	< 0.0001
Height (cm)	165.1 (6.7)	153.3 (5.5)	< 0.0001
**Body mass index **(kg/m^2^)	22.7 (3.0)	22.3 (3.5)	0.029
Lumbar spine BMD (g/cm^2^)	0.93 (0.14)	0.87 (0.15)	< 0.0001
Femoral neck BMD (g/cm^2^)	0.75 (0.15)	0.67 (0.12)	< 0.0001
Total hip BMD (g/cm^2^)	0.94 (0.15)	0.84 (0.13)	< 0.0001
Whole body BMD (g/cm^2^)	1.06 (0.1)	0.99 (0.11)	< 0.0001

Values are mean (standard deviation)

BMD, bone mineral density

*P*-value was derived from unpaired t-test for difference between men and women.

### Peak bone mineral density

**Figure 1:**
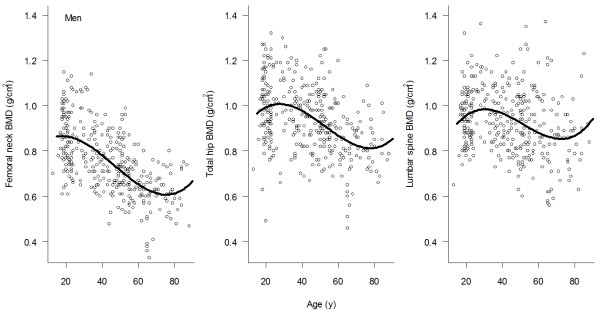
**Relationship between age and bone density at the femoral neck, total hip, and lumbar spine for men**.

**Figure 2:**
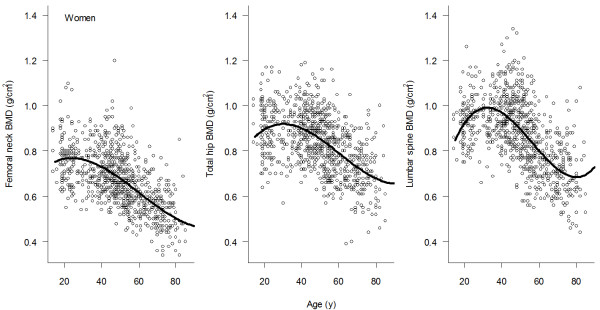
**Relationship between age and bone density at the femoral neck, total hip, and lumbar spine for women**.

The relationship between BMD and age was best described by the third-degree polynomial regression model (Figures 1 and 2). The relationship was characterized by three phases, namely, BMD increased between the ages of 18 and 25, followed by a steady period (aged between 25 and 45), and then gradually declined after the age of 45. The age-related decrease in BMD in women was greater than that in men. For example, compared with lumbar spine BMD among women aged between 20-30 years, lumbar spine BMD among women aged 70+ years was decreased by 27%; however, in men, the corresponding rate of decrease was ~15%. A similar sex-differential decline was also observed in femoral neck BMD.

Based on the parameters of the polynomial regression model (Table 2), we estimated *p*BMD and the age of *p*BMD for men and women (Table 3). Consistently, *p*BMD was higher in men compared with women, but the difference was dependent on skeletal site. For example, for lumbar spine, *p*BMD in men (1.05 ± 0.12 g/cm^2^; mean ± SD) was about 9% higher than in women (0.96 ± 0.11 g/cm^2^). Similarly, *pBMD *at the femoral neck in men (0.85 ± 0.13 g/cm^2^) was 6% higher than in women (0.80 ± 0.11 g/cm^2^). The age achieving *p*BMD was reached in women was younger than in men. For example, at the femoral neck, age of *p*BMD in women was 22.4 years (95% CI: 19 - 24) which was earlier than in men (26; 95% CI: 24 - 29). This trend was also observed at the lumbar spine (25 in women and 27 years in men).

**Table 2:** Estimates of parameters of the third degree polynomial regression model

Bone mineral density	Parameters			R^2^	SEE
			
	β_1 _age	β_2 _age^2^	β_3 _age^3^		
**Femoral neck**					
Men	0.01098	-0.00038	0.0000027	0.38	0.11
	(0.00698)	-0.00015	-0.000001		
Women	0.01119	-0.00029	0.0000016	0.38	0.1
	(0.0044)	(0.00009)	(0.0000006)		

**Total hip**					
Men	0.0198	-0.0005	0.000003	0.18	0.13
	(0.0082)	(0.0001)	(0.000001)		
Women	0.0194	-0.0004	0.000002	0.29	0.11
	(0.005)	(0.0001)	(0.0000007)		

**Lumbar spine**					
Men	0.0228	-0.00053	0.000003	0.08	0.14
	(0.0082)	(0.00018)	(0.000001)		
Women	0.0423	-0.0009	0.000005	0.39	0.12
	(0.0053)	(0.0001)	(0.0000008)		

Values are coefficient (standard error) of the model BMD = α + β_1_age + β_2_age^2 ^+ β_3_age^3^

R^2^, coefficient of determination indicates the proportion of variance in BMD that could be "explained" by the polynomial model; SEE, Standard error of estimate.

**Table 3:** Peak bone mineral density (pBMD) and age of pBMD in men and women

	pBMD (g/cm^2^)^a^	Age of pBMD (years)^b^
**Men**		
Femoral neck	0.85 (0.13)	26 (24 - 29)
Total hip	1.00 (0.13)	32 (29 - 35)
Lumbar spine	1.05 (0.12)	27 (25 - 29)

**Women**		
Femoral neck	0.80 (0.11)	22 (19 - 24)
Total hip	0.95 (0.12)	27 (25 - 30)
Lumbar spine	0.96 (0.11)	27 (25 - 29)

Values are ^a^mean (standard deviation) and ^b^mean (95% confidence interval)

### Prevalence of osteoporosis

Based on *pBMD *and *SD*, *T*-scores were calculated for men aged 50+ years or post-menopausal women, and these were referred to as *T*_VN_, to differentiate with *T*_DXA _which was automatically provided by the densitometer (Table 4). In women aged over 50 years, *T*_VN _was higher than *T*_DXA _at femoral neck (-1.84 ± 0.96 vs -2.27 ± 0.96; *P *< 0.0001) and at the lumbar spine (-1.61 ± 1.28 vs -2.39 ± 1.31; *P *< 0.0001). In men aged over 50 years, the same trend also was also found at the femoral neck (-1.50 ± 0.90 vs -2.01 ± 0.86; *P *< 0.0001), and at the lumbar spine (-1.33 ± 1.33 vs -1.81 ± 1.43; *P *< 0.0001).

**Table 4:** Prevalence of osteoporosis and osteopenia in men and women aged 50+ years

	Men		Women	
	
	*T*_DXA_	*T*_VN_	*T*_DXA_	*T*_V_
**Femoral neck **				
Normal	14 (10.4)	43 (31.8)	41 (10.1)	76 (18.8)
Osteopenia	81 (60.0)	78 (57.8)	187 (46.2)	213 (52.6)
Osteoporosis	40 (29.6)	14 (10.4)	177 (43.7)	116 (28.6)

**Total hip**				
Normal	NA	66 (48.9)	NA	141 (34.8)
Osteopenia	NA	61 (45.2)	NA	190 (46.9)
Osteoporosis	NA	8 (5.9)	NA	74 (18.3)

**Lumbar spine**				
Normal	37 (27.4)	129 (31.9)	62 (15.3)	49 (36.3)
Osteopenia	56 (41.5)	159 (39.3)	127 (31.4)	62 (45.9)
Osteoporosis	42 (31.1)	117 (28.9)	216 (53.3)	24 (17.8)

Data are actual number of individuals in each subgroup, and percentage of sex-specific total.

As expected, although absolute values of *T*_VN _and *T*_DXA _were different, the correlation between them was high (*r *> 0.98). The linear equation (without intercept) linking the two scores is as follows: at the femoral neck, *T*_VN _= 1.177 × *T*_DXA _for women, and *T*_VN _= 1.246 × *T*_DXA _for men; at the lumbar spine, *T*_VN _= 1.298 × *T*_DXA _for women, and TVN = 1.207 × T_DXA _for men. The equations suggest that, for example, at the femoral neck, *T*_VN _was higher than *T*_DXA _by 0.18 *SD *(in men) to 0.25 *SD *(in women).

The concordance between two diagnoses of osteoporosis (i.e., *T*_VN _and *T*_DXA_) is shown in Table 5. *T*_DXA _tended to over-diagnose osteoporosis more than did *T*_VN_. In women aged 50+ years, using femoral neck *T*_VN_, the prevalence of osteoporosis was 28.6%, but when the diagnosis was based on *T*_DXA_, the prevalence was 43.7%. In men aged 50+ years, the *T*_VN_-based prevalence of osteoporosis was 10.4%, which was only a-third of the *T*_DXA_-based prevalence (29.6%). The discrepancy mainly occurred in the osteopenic group. For example, among 40 men diagnosed by *T*_DXA _to have osteoporosis, there was 65% of them (*n = *26) were actually identified as having osteopenia by *T*_VN_. Similarly, among 177 women where were diagnosed with osteoporosis by *T*_DXA_, 35% (*n = *61) were actually osteopenic by *T*_VN_. The kappa statistic was 0.54 for women and 0.41 for men.

**Table 5:** Concordance in diagnosis of osteoporosis between DXA provided T-scores and actual T-scores

Diagnosis based on	Diagnosis based on femoral neck *T*_VN_		
	
femoral neck *T*_DXA_	Normal	Osteopenia	Osteoporosis
**Men**			
Normal	14 (100.0)	0	0
Osteopenia	29 (35.8)	52 (64.2)	0
Osteoporosis	0 (0.0)	26 (65.0)	14 (35.0)

**Women**			
Normal	40 (97.6)	1 (2.4)	0
Osteopenia	36 (19.4)	151 (80.6)	0
Osteoporosis	0	61 (34.5)	116 (65.5)

Values are shown as number of individuals in each subgroup, and percentage of row-wise total. Kappa statistic for men: κ = 0.41 (95% CI: 0.30 - 0.52); women: κ = 0.54 (95% CI: 0.48 - 0.60)

Using the National Health and Nutrition Examination Survey (NHANES) reference data for US Whites (aged between 20 and 29) [13], we computed *T*-score for each individual aged 50+ years, and classified into either normal, osteopenia or osteoporosis group. We found that the prevalence of osteoporosis was 30% (n = 40/135) in men and 43% (n = 160/368) in women. These prevalence rates are almost identical to the prevalence derived from the *T*_DXA_. In fact, the concordance in osteoporosis classification between *T*_DXA _and NHANES data was 100% for men and 96% for women.

## Discussion

To assess the magnitude of the problem, it is essential to establish an appropriate measure for the diagnosis of osteoporosis. Currently, osteoporosis is operationally defined in terms of BMD, which is compared to a normative database [5]. However, it is well known that measured values of BMD differ across ethnicities [9,11,14], and the referent database should therefore be ethnicity-specific. In this study, we have shown that there was a considerable discrepancy in the diagnosis of osteoporosis between referent data derived from the local population and referent data that are provided by the densitometer.

It is clear from this analysis that the densitometer reference data over-diagnosed osteoporosis in the Vietnamese population. Using the local normative data, we found that the prevalence of osteoporosis in Vietnamese women and men aged 50+ years was 29% and 10%, respectively. However, using the DXA-provided normative data, the prevalence in women and men was 44% and 30%, respectively. The discrepancy raises a question of which T-score is more appropriate. In a recent study in 328 Vietnamese postmenopausal women using DXA Lunar Prodigy, the prevalence of osteoporosis was 26% [15]. Another smaller study in Vietnamese postmenopausal women living in United State showed that this prevalence was 37% [16]. The prevalence of osteoporosis in postmenopausal Thai women was around 29% [17]. In Caucasians, the prevalence of osteoporosis in postmenopausal women ranged between 20% and 25% [18,19]. In summary, most of these studies in Asian and Caucasian women found that the prevalence of osteoporosis ranged between 20 and 30% [18-20], which is highly consistent with the present study's estimate. These data also suggest that the densitometer-provided *T*-score is not appropriate for the diagnosis of osteoporosis in Vietnamese women.

Why there were differences between *T*_VN _and *T*_DXA_? The most "proximate" explanation is that there were differences in peak BMD and standard deviation between the Hologic normative data and the present normative data. However, the standard deviation in BMD is very stable across populations; therefore, the main reason could be that peak BMD value provided by the Hologic densitometer was higher than peak BMD in Vietnamese. Assuming that SD of femoral neck BMD was 0.12 g/cm^2^, with *T*_DXA_, one could infer that peak BMD was 0.92 and 0.86 g/cm^2 ^in men and women, respectively. These values are identical to the femoral neck BMD reference values for US White men and women of the National Health and Nutrition Examination Survey (NHANES)[13]. In reality, the observed peak BMD in our study was 0.85 g/cm^2 ^(men) and 0.80 g/cm^2 ^(women). It is obvious that the peak BMD provided by the densitometer was derived from a non-Vietnamese population, which may not be applicable to the Vietnamese population.

In this study, the relationship between BMD and age followed a third degree polynomial function, which is consistent with a recent study [15]. According to this functional relationship, Vietnamese women achieved their peak BMD at the age of 27-29, which was later than that in Caucasian (20-25 years). Although it is not possible to determine the underlying factors for this apparent difference, it is well-known that Asian girls tend to have a later menarche than Caucasian girls (13 vs 12 years).

Osteoporosis in men, particularly Asian men, has not been well documented. The present study was among the first research about osteoporosis in Asian men. In this study, about one tenth of men aged over 50 had osteoporosis. This prevalence is highly comparable with previous estimate from Caucasian men [19]. Individuals with osteoporosis are at high risk of fragility fracture [21,22]. In this study, we found that almost 30% of women (and 10% of men) aged 50+ years had osteoporosis, implying that the magnitude of osteoporosis in Vietnam is as high as in developed countries.

The present results have to be interpreted within the context of strengths and potential limitations. First, the study represents one of the largest studies of osteoporosis in Asian populations, and as such, it increased the reliability of estimates of peak bone mass and prevalence of osteoporosis. Second, the study population is highly homogeneous, which reduces the effects of potential confounders that could compromise the estimates. The participants were randomly selected according to a rigorous sampling scheme, which ensures the representativeness of the general population. Third, the technique of measurement of BMD is considered "gold standard" for the assessment of bone strength. Nevertheless, the study also has a number of potential weaknesses. The participants in this study were sampled from an urban population; as a result, the study's finding may not be generalizable to the rural population. Because we excluded individuals with diseases deemed to interfere with bone metabolism, the prevalence of osteoporosis reported here could be an underestimate of the prevalence in the general population. Ideally, peak bone density should be estimated from a longitudinal study in which a large number of men and women is followed from the age of 5 till the age of 30, but such a study is not practically feasible. On the other hand, estimate of peak BMD in cross-sectional study such as the present study can be biased by unmeasured confounders.

Nevertheless, the present findings have important public health and clinical implications. Because individuals with T-scores being or less than -2.5 are often treated, the over-diagnosis by *T*_DXA _could have led to over-treatment in the general population. Moreover, individuals with T-scores being or less than -2.5 are also candidates for anti-fracture clinical trials or clinical studies, the use of *T*_DXA _could have included some women in such studies and exposed them to unnecessary risk. Thus, it seems prudent to use local normative data for the diagnosis of osteoporosis in order to avoid over-diagnosis or over-treatment.

## Conclusion

In summary, these data suggest that the prevalence of osteoporosis in Vietnamese men (10%) and women (30%) aged 50+ years is comparable with those in Caucasian populations. The data also indicated that the *T-*score provided by the Hologic QDR4500 over-diagnosed osteoporosis in Vietnamese men and women. We propose to use the data developed in this study for the diagnosis of osteoporosis in the Vietnamese population.

## Competing interests

All authors declare that they have no competing interests with regard to this work. Professor T. Nguyen received honorarium for speaking and providing consultant services to MSD Vietnam Ltd, Sanofi-Aventis, Norvatis, and Roche.

## Authors' contributions

Contributions of the authors to the manuscript included *Study concept and design: *LHP and TVN; *Acquisition of data: *LHP, UDTN, HNP. *Analysis and interpretation of data: *LHP, NDN, TVN; *Drafting the manuscript: *LHP, TVN, NDN; *Statistical analysis: *NDN, LHP, TVN; *Critical revision of the manuscript: *LHP, TVN, NDN, UDTN, HNP. All authors read and approved the final manuscript.

## Pre-publication history

The pre-publication history for this paper can be accessed here:

http://www.biomedcentral.com/1471-2474/12/182/prepub
